# Ex vivo investigation on the effect of minimally invasive endodontic treatment on vertical root fracture resistance and crack formation

**DOI:** 10.1038/s41598-024-63396-y

**Published:** 2024-06-08

**Authors:** Andreas Rathke, Henry Frehse, Maria Bechtold

**Affiliations:** 1 Faculty of Medicine, University of Ulm, Albert-Einstein-Allee 7, 89081 Ulm, Germany; 2 Dentsply Sirona, DeTrey-Strasse 1, 78467 Konstanz, Germany; 3 Private Practice, Münchener Straße 1, 82362 Weilheim, Germany

**Keywords:** Chewing simulation, Dentin crack, Minimally invasive endodontics, Root canal shaping, Sealer, Vertical root fracture, Microscopy, Dentistry

## Abstract

The evidence base on minimally invasive endodontic (MIE) treatment is limited. This study investigated the influence of MIE shaping on vertical root fracture (VRF) resistance and crack formation of root canal filled teeth. Human maxillary central incisors were randomized into six groups (*n* = 18, power = 0.9) and embedded in acrylic blocks with artificial periodontal ligaments. The root canals were either instrumented to size #40 and 0.04 taper (+MIE) or enlarged to ISO size #80 (−MIE). The canals were filled with cement-based (C) or adhesive resin-based (A) sealers in single-cone technique. The controls received no treatment or were left unfilled. After chewing simulation (staircase method, 25–150 N, 120,000×), the crack formation on the root surface was analyzed using stereomicroscope/digital imaging and classified (no defect, craze line, vertical crack, horizontal crack). Subsequently, the samples were loaded until fracture. The incidence of defects (56% vertical cracks) was not significantly different between the groups (*p* ≥ 0.077). VRF resistance was significantly higher in untreated teeth than in +MIE/C (*p* = 0.020) but did not significantly differ between the other groups (*p* ≥ 0.068). Minimal canal shaping did not reduce the risk of vertical root fracture and defects of root canal filled teeth.

## Introduction

The main objectives of endodontic treatment are the removal of infected hard and soft tissue, the disinfection of the root canal system and its canal shaping for obturation, usually with gutta-percha (GP) and root canal sealer^1,2^. Long-term survival rates of 86% after 20 years have been reported for root canal filled teeth^3^, although clinical success varied widely depending on several factors^4^. The reasons for failure included persistent or recurrent infections^5^, but also root fractures such as vertical root fracture (VRF)^6^. Several iatrogenic and non-iatrogenic factors, such as dentin removal due to the coronal access cavity, root canal shaping and post placement, pressure during obturation or altered dentin properties, have been associated with the development of VRF^6^. Subcritical cracks could propagate under chewing forces or traumatic overloading into VRF, often requiring tooth extraction^6^. The prevalence of VRF in root canal filled teeth has been reported in the literature to be between 3 and 32%^6–8^.

Several authors have suggested that minimally invasive endodontic (MIE) treatment, which involves the use of a small access cavity and minimal canal shaping, as well as adhesive resin-based approaches, increases the fracture resistance of root canal filled teeth^9–12^. However, smaller access preparations increased the difficulty of cleaning, disinfection and obturation^13,14^ and did not necessarily improve the fracture susceptibility of endodontically treated teeth^14,15^. In cases that could be adhesively restored with composite resins, there was no significant difference in fracture resistance between teeth treated with traditional straight-line and minimally invasive access cavities^16^. The important role of adhesive restorative techniques in coronal reinforcement has been highlighted^12,16^.

Canal shaping should also be as conservative as practical for preserving root dentin, respecting the anatomical canal shape and preventing root perforation^1,2,10^. Potential preparation-related root weakening could be reinforced by adhesive resin-based obturation^17–19^, but long-term studies have shown significantly lower success rates than obturation with GP and sealers^20,21^. To reduce the risks of crack formation and root fracture, authors instead recommended MIE shaping with smaller file sizes (in the range between #20 and #40 in combination with 0.04 to 0.06 tapers), depending on the canal morphology^9,10,22^. However, insufficient canal debridement could lead to treatment failure, particularly in teeth with infected and necrotic pulp. Arguments were made for better cleaning and disinfection of canals with larger file sizes in combination with 0.02 to 0.05 tapers^1,9,23^, even when activated irrigation strategies were used^2^. In this regard, larger file tapers could not compensate for smaller file sizes^24^. However, it remains controversial whether the selected file size and taper influence fracture resistance^9,22,25,26^ and crack formation^22,27^. The latest attempts to systematically review results from in vitro studies to answer the question of whether MIE shaping increases the fracture resistance of root canal treated teeth or not were inconclusive^28^. Additionally, the clinical evidence on MIE treatment is limited^2,10,11^. For dental practitioners, this is not a satisfactory result.

The majority of in vitro studies analyzed root fractures caused by compressive loading^29^. Only a few authors have focused on compressive loading of root canal treated teeth after fatigue loading^26,30–32^, which provides better clinical insight. Therefore, by testing compressive fracture loads after chewing simulation, this study aimed to investigate the influence of MIE shaping on resistance against VRF and dentin defects of root canal filled teeth using different obturation materials. The null hypothesis was that there would be no difference in VRF resistance and crack formation between the experimental groups.

## Methods

### Sample size calculation

Based on previous investigations^32^, the sample size was calculated using these data and two-sided Welch's t-test for unequal variance at a significance level of *α* = 0.05 and a power of 0.9 (nQuery Advisor version 7; Statistical Solutions, Cork, Ireland). The sample size was evaluated as *n* = 15 for each group. Considering possible dropouts and a deviation of normality assumptions, a sample size of *n* = 18 was used in the study.

### Sample selection and preparation

Extracted human teeth were collected from dentists and dental clinics for reasons not related to this study and stored in 1% chloramine-T solution (University Pharmacy, Ulm, Germany). All the donors were adults and provided written informed consent for research purposes. The teeth used were irreversibly anonymized and not traceable. In accordance with the German regulations of the central ethical committee for the use of human body material in medicine^33^ and the local ethics committee of the University of Ulm, no ethical approval was mandatory for these samples and this type of study. Permanent maxillary central incisors with a single, straight root canal and complete root formation were selected. Teeth were cleaned with scalers, and crowns were removed using a diamond saw at slow speed (WOCO 50/Med; Conrad, Clausthal-Zellerfeld, Germany) to obtain a standardized root length of 13 mm. A stereomicroscope (Stemi SV8; Zeiss, Oberkochen, Germany) at 12× magnification was used to exclude teeth with caries, restorations, root fillings, resorptions, or pre-existing dentin defects. The inclusion and exclusion criteria were consistent with previous studies^25,27,32,34^. After the teeth were numbered, cross sections of the roots were measured at the level of the cutting surface in the mesio-distal and bucco-palatal directions with a digital caliper (Garant; Hoffmann, Munich, Germany). The area of the ellipsed root cross section (A) was calculated as A = *π* ÷ 4 × a × b (where a and b were the mesio-distal and bucco-palatal dimensions, respectively, in mm). Extremely small or large root cross-sections were excluded. The remaining samples were randomized into six groups (two control and four experimental groups) of 18 roots each using a randomization software (ROM; Institute of Epidemiology and Medical Biometry, University of Ulm, Germany)^35^. No significant differences were found between the groups regarding the mean [SD] cross-sectional area (35.7 [3.6] mm^2^; *p* > 0.05, one-way ANOVA). To simulate the periodontal ligament with relatively uniform stress distribution, the roots were wrapped in one layer of latex rubber milk (Suter Kunststoffe; Jegenstorf, Switzerland) with a thickness of approximately 250 µm and embedded in acrylic resin (Technovit 4071; Heraeus Kulzer, Hanau, Germany) with the cervical root third being exposed.

### Root canal treatment

In the negative control, the root canals were left untreated. Endodontic treatments were performed by a single operator with (+) or without (−) MIE shaping. Canal patency was controlled with ISO size #10 hand files (K-file; Kerr, Orange, CA, USA). The working length was set to 12 mm, and K-files up to ISO size #20 were used to create a glide path. Canals in the +MIE group were instrumented with nickel-titanium (NiTi) rotary files (Twisted File; Kerr) using the single-length technique in the file sequence of size #25, size #30, and size #35 in combination with 0.06 tapers up to size #40 and 0.04 taper. The files were rotated with a 4:1 reduction handpiece (WD-77 M; W&H, Buermoos, Austria) powered by a torque-control motor (Endo IT professional; VDW, Munich, Germany). During instrumentation, the canals were irrigated with 5 ml of 3% sodium hypochlorite (NaOCl) solution (University Pharmacy, Ulm, Germany), and 15% ethylenediaminetetraacetic acid (EDTA) chelating agent (Glyde File Prep; Dentsply Sirona, Ballaigues, Switzerland) was used to remove the smear layer. After a flush with 5 ml of distilled water, the canals were dried with paper points and filled with non-adhesive calcium hydroxide-based (C) (Sealapex; Kerr) or adhesive resin-based (A) (RealSeal SE [RS]; Kerr) sealers in single-cone obturation technique. The sealers were mixed according to the manufacturer’s instructions, placed with a lentulo and filled with the matched Twisted File GP or RS cones (Kerr). Canals in the −MIE group were instrumented as those in the +MIE group and then enlarged with Twisted File size #50 and 0.04 taper, followed by manual widening with K-files from ISO size #60 and ISO size #70 to ISO size #80. During instrumentation, the canals were irrigated with 5 ml of 3% NaOCL and 15% EDTA. After a flush with 5 ml of distilled water and drying with paper points, the canals were either left unfilled (positive control) or filled according to +MIE. The 1-mm-deep canal orifices were filled with a temporary filling material (Cavit; 3M Espe, Seefeld, Germany).

### Chewing simulation and VRF testing

After storage in water for 24 h at 37 °C, the samples were subjected to 1500 thermocycles in distilled water at 5–55 °C with a dwelling time of 20 s in each bath and a transfer time of 5 s (Haake W15; Willytec, Gräfelfing, Germany). Mechanical loading was performed according to the staircase method starting at a load of 25 N at an angle of 10° to the axial direction of the roots in a chewing simulator (Standard 2002; Willytec)^32^. Every 20,000 cycles at a frequency of 2 Hz, the load was increased in increments of 25 N until 120,000 cycles were reached. The 1-mm-unfilled canal space ensured that the force applied by the coneshaped metal antagonist at an angle of 120° was transmitted to the root dentin rather than to the root canal filling. The diameter of the truncated cone was dimensioned in such a way that the metal tip fitted exactly into the canal space.

**Figure 1:**
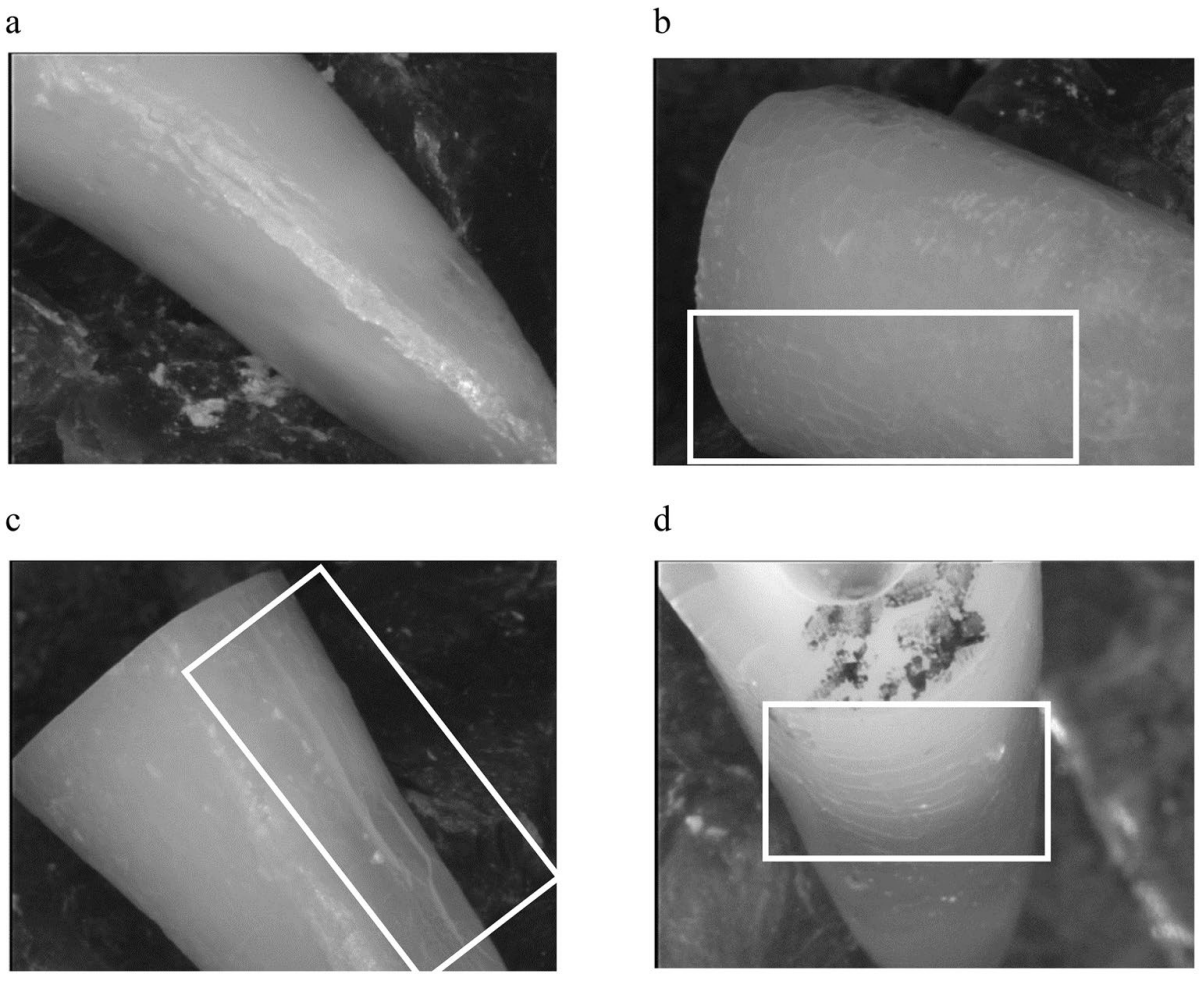
Representative images of the different defect modes along the outer root surface after chewing simulation. (**a**) No dentin defect, (**b**) craze line, (**c**) vertical root crack, (**d**) horizontal root crack. Original magnification: 12-fold.

VRF resistance and crack formation were determined from the samples that survived chewing simulation. Pre-testing failures (PTFs) were recorded. The external root surfaces were examined under the stereomicroscope using a cold light source (Stemi SV8; Zeiss). Because of the latex milk, the roots could be removed from the acrylic blocks. Digital images were captured under 12–100× magnification using a digital camera (3CCD Color Video Camera; Sony, Tokyo, Japan) attached to the stereomicroscope. Crack formation was analyzed per root third (cervical, middle, apical) as follows: (a) no defect, (b) craze line, (c) vertical crack, and (d) horizontal crack. Representative images of the defect patterns are shown in Fig. 1. Different defect patterns in the same root third were recorded, resulting in a maximum of nine defects per root. After microscopic analysis, the roots were reinserted to the acrylic blocks and subjected to VRF testing. The same antagonist as used for the chewing simulator was attached to the load cell of a universal testing machine (Zwicki 1120; Zwick, Ulm, Germany). The samples were loaded until fracture with a crosshead speed of 1 mm/min. The fracture load (N) was recorded when the force in the load-strain curve decreased by 30%.

### Statistics

Statistical analysis was performed with the aid of a statistical software (IBM SPSS version 19 for Windows; IBM, Armonk, NY, USA). The significance level was set in advance at *α* = 0.05. As the Shapiro–Wilk test indicated that the VRF resistance (*p* = 0.002) and crack formation (*p* = 0.0001) data were not normally distributed, differences between the groups were compared with the nonparametric Kruskal–Wallis test. Post-hoc multiple comparisons were performed using the Mann–Whitney U test with Bonferroni correction for 15 two-group comparisons.

## Results

A total of seven PTFs were recorded. Two samples each from the negative control (untreated teeth) and the +MIE/A and −MIE/A groups as well as one sample from the +MIE/C group showed visible fractures after chewing simulation. The VRF resistance and crack formation of the surviving samples are presented as medians with interquartile ranges in Table 1. The results of the Kruskal–Wallis test indicated significant intergroup differences in the incidence of dentin defects (*p* = 0.006). However, post-hoc multiple comparisons did not reveal statistical evidence for a significant difference between the groups (*p* ≥ 0.077). Minimal shaping (+MIE/A) resulted in the lowest number of dentin defects (1 [0–2]), while the positive control (without obturation) caused the highest incidence of defects (3 [2–4]). Among the groups, 31.7% of the samples showed dentin defects in the apical root third, while 63.4% and 73.3% of the samples had defects in the cervical and middle root sections, respectively. The majority of the dentin defects were vertical root cracks (56.3%), followed by horizontal root cracks (23.6%) and craze lines (20.1%).

**Table 1:**
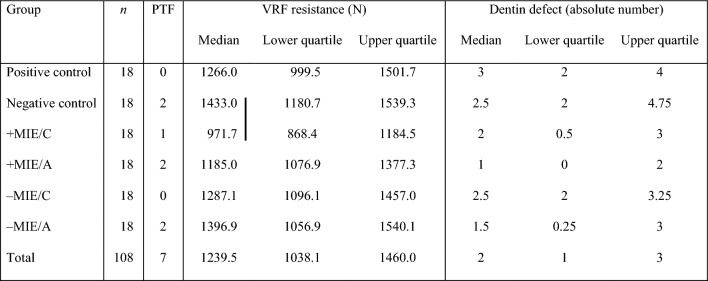
Median values with interquartile ranges of VRF resistance (N) and incidence of dentin defects (absolute number) in the different groups.

*A* adhesive resin-based sealer (using single-cone obturation), *C* cement-based sealer (using single-cone obturation), *MIE* minimally invasive endodontic shaping, *Negative control* untreated teeth*, Positive control* without obturation, *PTF* pre-testing failure, *VRF* vertical root fracture. Groups connected by vertical line are significantly different at *p* < 0.05.

VRF resistance was significantly different between the groups (*p* = 0.008). Post-hoc analysis revealed that VRF resistance was significantly higher in untreated teeth than in those in the +MIE/C group (*p* = 0.020). No significant difference was shown between the other groups (*p* ≥ 0.068). Among the experimental groups, +MIE/C had the lowest resistance to VRF (972 N [868–1185]), while −MIE/A had the highest resistance to VRF (1397 N [1057–1540]).

## Discussion

The present study showed that root canal filled teeth with minimally shaped root canals do not differ significantly from their more invasively prepared counterparts in terms of vertical root fracture (VRF) resistance and crack formation, irrespective of the obturation material used. Therefore, the null hypothesis could not be rejected.

In many studies, fracture resistance measurements and analyses were carried out on root canal treated teeth that had been decoronated^19,32,34^. Studies have shown that the differences in fracture resistance between teeth with and without a minimally invasive access cavity are too small to be relevant^14–16^. The teeth in this study were loaded without considering the access cavity to avoid confounding effects such as cuspal deflection and coronal reinforcement by adhesive restoration and to evaluate only potential radicular reinforcement. A multifactorial study design, consisting of the factors minimally invasive endodontic (MIE) shaping and obturation material, was used to investigate the outcome parameters VRF resistance and crack formation. The data could not support the intuitively obvious theory that teeth treated with MIE shaping are less susceptible to fracture^9–12^. Finite element method (FEM) analysis of a maxillary central incisor revealed higher radicular stresses during loading when the root canal was prepared to larger diameters^36^. Fracture load data of maxillary central incisors confirmed a significant positive correlation between canal enlargement and fracture susceptibility^37,38^, whereas no such correlation was found in another FEM study using root dentin sections^39^. More recently, combined experimental and FEM analyses have shown that the fracture load of root dentin sections increases with larger diameter of instrumented canals^40^. This could be because the circumferential area for stress distribution increases with canal enlargement^40^. However, the stress distribution was less uniform when the canal shape was oval, resulting in stress concentration areas^41^. Other factors, such as the root morphology, the taper of the canal and its curvature, also influenced the fracture susceptibility^40,41^.

For sample selection, only maxillary central incisors of comparable length and cross-section and relatively straight canals were used. Stratified randomization of the root size using randomization software^35^ ensured standardization of the samples to avoid potential selection bias. The diameter at the orifice level was approximately 1 mm in all canal preparations, while the apical canal diameter of the more invasive shaping was almost twice that of the MIE shaping. A wide range of apical canal diameters has been reported^9^, and files up to size #80 were used for maxillary central incisors^37,38^. Apical enlargement has been recommended for these teeth to remove bacteria and infected dentin in the oval canals^1^. For sufficient debridement in the buccolingual direction, a hybrid technique combining rotary NiTi files with conventional hand files has been proposed^1^, which was also used in this study. FEM analysis suggested that eliminating stress-increasing areas such as those in the buccal and lingual recesses of oval canals reduces tensile stress in root dentin^41^. On the other hand, the use of larger and stiffer K-files may have resulted in more instrumentation stress on the root canal wall, especially at the narrow mesiodistal diameter of the canal. This could explain the higher, albeit not significant, incidence of dentin defects compared to MIE shaping.

The shaped canals were filled in single-cone technique to reduce the potential risk of dentin defects during obturation^34^. It was assumed that the filling forces are lower than those of other obturation techniques that exert compaction forces on the canal wall^34^. In the present study, the obturation materials under investigation performed equally. No reinforcement of the root canal filling was observed compared to that of the positive control (without obturation), in contrast to the findings of other authors, who confirmed that the use of obturation materials can increase the fracture resistance of root canal filled teeth^18,19^. Flexural properties such as the tensile strength and elastic modulus of obturation materials were found to be too low compared to those of root dentin to reinforce teeth^17,38^. Notably, a calcium hydroxide-based sealer with inferior bond strength, marked solubility, and limited durability in root canals was used as a representative non-adhesive sealer to investigate the potential radicular reinforcement of the adhesive resin-based sealer^42^. Adhesive and self-adhesive resin-based sealers have been suggested to bond to the root canal^17,18^. However, it has been shown extensively that intracanal bonding is compromised, for example, due to the high configuration factor in the root canal and the associated polymerization stress^17,42^, which may lead to adhesive failure and disintegration of the obturation during clinical service^20,21^. Given the limitations of the two sealers tested, future studies should include contemporary sealers with enhanced material characteristics and clinical performance to provide a more valid representation of the effect of obturation.

In the present study, a non-destructive examination of the root surfaces was performed using stereomicroscope/digital imaging. Optical microscopy has proven to be well suited for detecting crack formation on the root surface of root canal treated teeth^27,43^, Another non-destructive technique that has been used in several studies is micro-computed tomography (micro-CT). A methodological study comparing four different imaging techniques on root canal treated teeth observed no significant difference between stereomicroscopy and micro-CT in detecting cracks on root dentin^43^. In the present study, the incidence of dentin defects decreased toward the apical third of the root, regardless of the group. Most of these dentin defects were vertical root cracks (56%). One of the possible reasons could be that direct loading of the root canal filling was avoided, which may have contributed to a stress reduction in the apical third. Instead, the load was transmitted to the root canal walls, which was more likely to cause vertical cracks and VRF due to the wedge effect^32^. Another explanation could be that the tubular density in the root canal decreases from the cervical to the apical region. At high tubule density, cracks propagated more frequently through the peritubular dentin, whereas at low tubule density, crack propagation was determined by the intertubular dentin. Fatigue analyses have shown that peritubular dentin is more mineralized than intertubular dentin and is more brittle and easier to crack^44^. In microtensile tests on maxillary anterior roots, a significantly lower tensile strength was measured in cervical dentin than in middle-apical dentin^45^. However, the present results may not be fully generalizable to the clinical setting. Although attempts have been made to simulate the clinical condition using artificial periodontal ligaments and chewing simulation, the biological structures and chewing forces in vivo are more complex. With the staircase method used, the force increased gradually for a limited number of cycles, whereas the number of cycles to root fracture is much higher under functional chewing force^6^. Furthermore, coronal reinforcement by crown/cuspal coverage or adhesive restoration could lead to a more favorable stress distribution in the cervical area of the root and the pericervical dentin.

The prospective power analysis indicated that significant results can be obtained with 18 samples per group. Three samples per group were prepared in case of possible processing errors. Seven of the original 108 teeth tested did not survive the chewing simulation and were rated as pre-test failures (PTFs). Investigators either assigned PTFs a fracture strength value of zero^30^ or discarded them after the chewing simulation^31^, as in this study. The rationale for exclusion was that two PTFs also occurred in untreated teeth for which consistently high fracture load values were reported and which therefore served as a negative control^18,32,34^. It has been reported that in mechanical engineering, approximately 10% of fatigued samples fail prematurely due to processing errors or accidental loading^29^. Fatigue failure of extracted human teeth is also influenced by other factors, such as differences in tooth age, dentin microstructure, and storage conditions^27,32^.

## Conclusions

Minimal canal shaping did not reduce the risk of vertical root fracture and incidence of dentin defects compared to the more invasively shaped counterparts, regardless of the obturation material used for root canal filling. When balancing the disinfection and shaping of root canal systems, clinicians should therefore be aware that minimally invasive shaping does not guarantee higher fracture resistance of root canal filled teeth.

## Data Availability

All the data underlying the results are available as part of the article, and no additional source data are applicable. The data presented in this study are available upon request from the corresponding author.
